# Late‐onset refractory hemolytic anemia in siblings treated for methionine synthase reductase deficiency: A rare complication possibly prevented by hydroxocobalamin dose escalation?

**DOI:** 10.1002/jmd2.12422

**Published:** 2024-04-15

**Authors:** Alexandre Nguyen, Samuel Deshayes, Marie Nowoczyn, Apolline Imbard, Lamisse Mansour‐Hendili, Alexandre Cesbron, Jean François Benoist, Manuel Schiff

**Affiliations:** ^1^ Department of Internal Medicine and Clinical Immunology Normandie Univ, UNICAEN, CHU Caen Normandie Caen France; ^2^ Biochemistry Laboratory CHU Caen Normandie Caen France; ^3^ Biochemistry Laboratory Necker University Hospital, APHP Paris France; ^4^ Département Médicaments et Technologies Pour la Santé (DMTS) Université Paris‐Saclay, CEA, INRAE, MetaboHUB Gif‐sur‐Yvette France; ^5^ LBM SeqOIA Paris France; ^6^ Department of Genetics Henri Mondor Hospital, APHP Créteil France; ^7^ Reference Center for Inherited Metabolic Diseases Necker University Hospital, APHP and University of Paris Cité Paris France; ^8^ INSERM UMRS_1163, Institut Imagine Paris France

**Keywords:** cblE, hemolysis, hemolytic anemia, hydroxocobalamin, LDH, methionine synthase reductase, MTRR, remethylation disorders

## Abstract

Methionine synthase reductase deficiency (cblE) is a rare autosomal recessive inborn error of cobalamin metabolism caused by pathogenic variants in the methionine synthase reductase gene (*MTRR*). Patients usually exhibit early‐onset bone marrow failure with pancytopenia including megaloblastic anemia. The latter can remain isolated or patients may present developmental delay and rarely macular dysfunction. Treatment mostly includes parenteral hydroxocobalamin to maximize the residual enzyme function and betaine to increase methionine concentrations and decrease homocysteine accumulation. We report herein 2 cblE siblings diagnosed in the neonatal period with isolated pancytopenia who, despite treatment, exhibited in adulthood hemolytic anemia (LDH >11 000 U/L, undetectable haptoglobin, elevated unconjugated bilirubin) which could finally be successfully treated by hydroxocobalamin dose escalation. There was no obvious trigger apart from a parvovirus B19 infection in one of the patients. This is the first report of such complications in adulthood. The use of LDH for disease monitoring could possibly be an additional useful biomarker to adjust hydroxocobalamin dosage. Bone marrow infection with parvovirus B19 can complicate this genetic disease with erythroblastopenia even in the absence of an immunocompromised status, as in other congenital hemolytic anemias. The observation of novel hemolytic features in this rare disease should raise awareness about specific complications in remethylation disorders and plea for hydroxocobalamin dose escalation.

**Synopsis**

To conclude, the observation of novel hemolytic and infectious features in this rare disease should raise awareness about specific adulthood complications in remethylation disorders to optimize management, especially hydroxocobalamin dose escalation.

## 1 INTRODUCTION

Cobalamin (Cbl, vitamin B12) is necessary for methylcobalamin and adenosylcobalamin production, the cofactors for methionine synthase (MTR) and methylmalonyl‐CoA mutase (Mut) respectively. Intracellular Cbl metabolism disorders can present with isolated hyperhomocysteinemia (HHC) or combined HHC with methylmalonic academia (MMA). cblC, cblD‐MMAHcy, cblF, cblJ present with combined HHC with MMA.^1^, ^2^ Isolated remethylation disorders include methionine synthase reductase deficiency (MTRR, cblE), methionine synthase deficiency (MTR, cblG), cblD‐Hcy, and Methylenetetrahydrofolate reductase (MTHFR) deficiency. Methionine synthase reductase (MTRR) is necessary to MTR in a stable complex to catalyze a reductive methylation of oxidized cobalamin (Cbl‐II) to methylcobalamin (MeCbl).^3^, ^4^ MTRR (cblE) patients usually exhibit symptoms in early childhood with failure to thrive, hypotonia, and megaloblastic anemia.^5^, ^6^ A few may present developmental delay, and cerebral atrophy with white matter abnormalities while others will only exhibit hematological abnormalities. Diagnosis is generally suspected because of elevated total plasma homocysteine and no MMA, before being confirmed by molecular analyses.^2^, ^7^ Patients are treated with hydroxocobalamin to maximize the residual enzyme function, betaine, and folinic acid to increase methionine concentrations and decrease homocysteine accumulation.^2^

We report herein 2 cblE siblings, diagnosed and treated in the neonatal period who presented during adulthood with severe refractory hemolytic anemia.

### 1.1 Case reports

**FIGURE 1:**
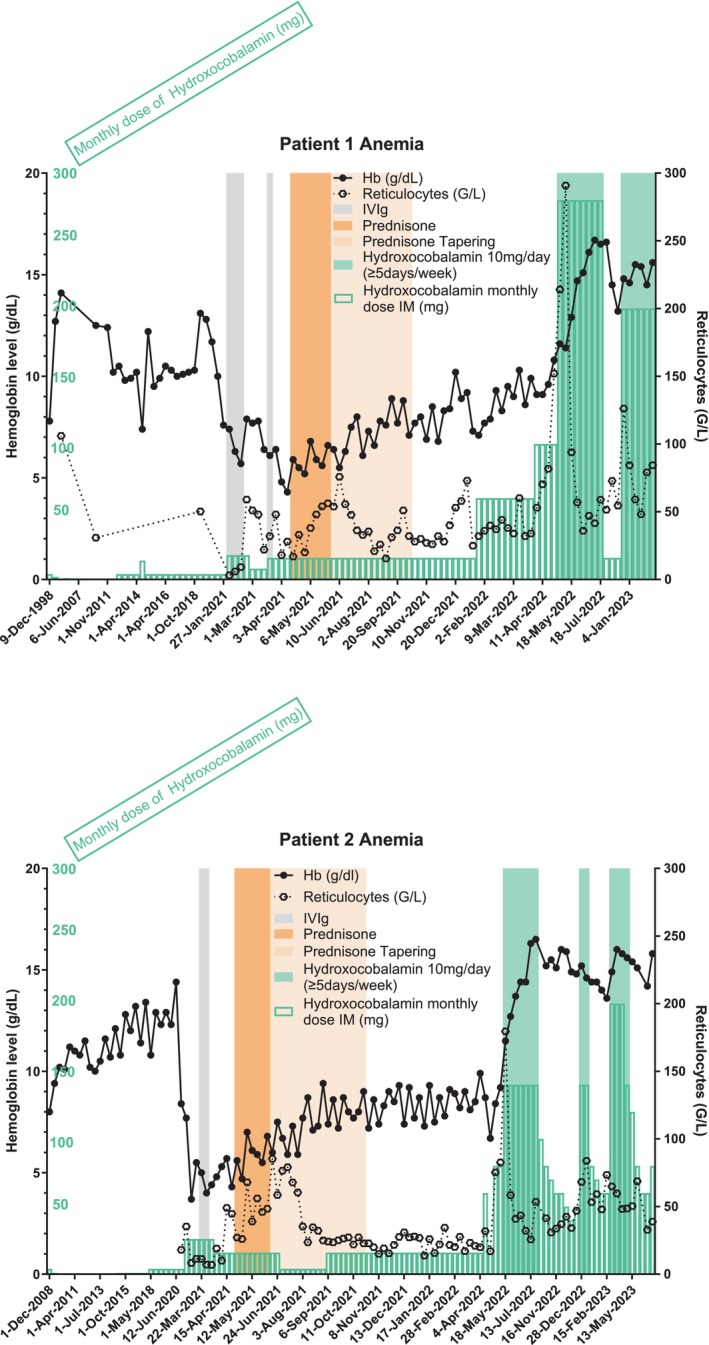
Anemia laboratory parameters evolution over time and changes in hydroxocobalamin dosages. Hb, hemoglobin concentration in g/dL; IVIg, intravenous immunoglobulins 1 g/kg/day; Prednisone, 1 mg/kg/day; Cumulative monthly dose of hydroxocobalamin is shown in green lined bars expressed in mg.

A 22‐year‐old Caucasian male was referred in February 2021 to our internal medicine and clinical immunology department for severe macrocytic anemia with a hemoglobin level at 6.3 g/dL, a major elevation of lactate dehydrogenase level (LDH) >11 000 IU/L, undetectable haptoglobin and a low reticulocyte count (6 G/L). He was affected with a remethylation disorder (methionine synthase reductase cblE) with compound heterozygote pathogenic variants in *MTRR* gene (c.1285 C>T, p.Gln429X and c.1910C>T, p.Ser637Leu), treated for many years with hydroxocobalamin 1 mg per week by intramuscular injection, folinic acid 5 mg/day, betaine 4.5 g 3 times a day, L‐methionine 200 mg 3 times a day, L‐carnitine 1 g 3 times a day. The patient was clinically reviewed twice a year and blood cell count was normal apart from chronic macrocytosis (Figure 1). The neonatal diagnosis had been made at 10 days old with first symptoms of pallor and megaloblastic anemia on the 4th day of life after an uncomplicated pregnancy and birth at 40 weeks from non‐consanguineous parents (already reported in Reference 8). At admission (22 years old) for this acute anemia, the treatment had been strengthened with intramuscular injection (IM) of hydroxocobalamin 1 mg daily for 2 weeks (Figure 1). Laboratory parameters confirmed hemolysis with LDH >11 000 IU/L (N: 135–225), undetectable haptoglobin (N: 0.3–2.0 g/L), free plasma hemoglobin at 0.13 g/dL (N: <0.05) and unconjugated hyperbilirubinemia at 23 μmol/L (N: <15.0 μmol/L). Reticulocyte count was very low at 9 G/L whereas the hemoglobin level was 6 g/dL (N: 13–5–17.5) with a high red blood cell volume of 112 fL (N: 78–98). There were no other cytopenias, no schistocytes, and no abnormal cells on the blood smear except some elliptocytes and dacryocytes. Thrombotic microangiopathy was ruled out due to the absence of schistocytes on multiple blood smears, arterial hypertension or other organs involvement such as kidneys or the nervous system. Serum folate concentration were normal at 35 nmol/L (N: 7–45) and vitamin B12 concentration was elevated >1100 pmol/L (N: 145–700). Direct antiglobulin test was negative for C3d, IgG, IgM, and IgA. The bone marrow aspiration only revealed dyserythropoiesis with asynchronism without any anomaly on granular, megakaryocyte, or lymphoid cells. PCR for parvovirus B19 (PVB19) was positive in the bone marrow, while negative in blood with a positive serology in IgM and IgG. Plasma methionine concentration was low‐normal at 18 μmol/L (N: 17–77) and plasma total homocysteine concentration was elevated at 126 μmol/L (N: <15 μmol/L), which were the patient's usual values in his medical follow‐up. Treatment with intravenous immunoglobulins 0.5 g/kg over 4 days was started for the hypothesis of PVB19 bone marrow infection, which was complicated with poor clinical tolerance due to fever, shivers, and myalgia. After treatment, LDH decreased to 5000 U/L but hemoglobin or reticulocyte levels were unchanged. Transient neutropenia and thrombocytopenia occurred. A red blood cell transfusion was administered before the patient was discharged with 2 mg of intramuscular hydroxocobalamin 3 times a week.

Only 3 weeks after being discharged, he was referred back for acute fatigue and anemia (hemoglobin at 5.6 g/dL). His 20‐year‐old brother also affected with cblE defect was also admitted for acute anemia (hemoglobin at 3.9 g/dL). Investigations did not find any trace of PVB19 using high sensitivity PCR detection for both brothers either in blood or bone marrow. In blood, there was IgG but no IgM anti PVB19 in the first bother and there was neither IgG nor IgM in the second brother. No environmental cause was found, especially potential MTR inhibitors, as they did not share the same roof or location, or the same food or hygiene products. Other hemolytic causes (paroxysmal nocturnal hemoglobinuria, parasites, lead, mercury or arsenic poisoning, other enzyme deficiencies such as glucose‐6‐phosphate dehydrogenase, pyruvate kinase deficiencies, Wilson's disease, modifications of treatments) were not found. Ektacytometry was compatible with their genetic disease. Erythropoietin concentration was highly elevated at 10 times the normal value. Hydroxocobalamin monthly dose escalations for the two brothers are shown in Figure 1 as green bars.

**FIGURE 2:**
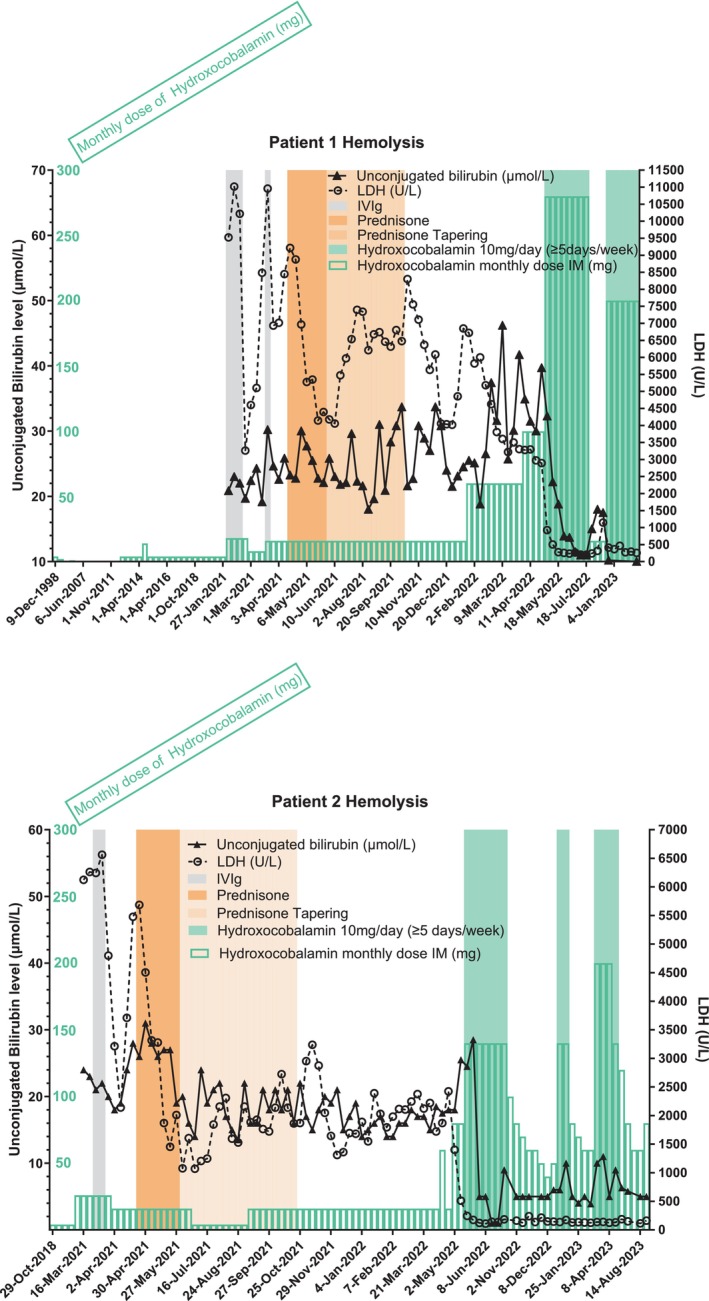
Hemolysis laboratory parameters evolution over time and changes in hydroxocobalamin dosages. LDH, lactate dehydrogenase concentration in international units/L; IVIg, intravenous immunoglobulins 1 g/kg/day; Prednisone, 1 mg/kg/day; Cumulative monthly dose of hydroxocobalamin is shown in green lined bars expressed in mg.

In both brothers, a higher dose of intramuscular hydroxocobalamin 3 mg 3 times a week and folinic acid 10 mg/day was administered for 10 months during advanced diagnostic testing, without any effect on hematological/metabolic parameters. Culture of erythroblast with serum inhibition test found no inhibition which ruled out auto‐immune erythroblastopenia. A therapeutic test was then initiated with prednisone 1 mg/kg/day for 1 month for both brothers, which allowed a decrease of LDH level to 1500 IU/L while remaining dependent on red blood cell transfusion once a week (Figure 2). For both brothers, genetic testing for red blood cell diseases and myelodysplastic syndromes did not find any anomaly on the usual targeted gene panels (Myeloid NGS panel CHU Caen, erythrocyte constitutional diseases NGS CHU Robert‐Debré). Whole genome sequencing was then performed and did not find any additional genetic anomaly other than their compound heterozygote status for MTRR.

**FIGURE 3:**
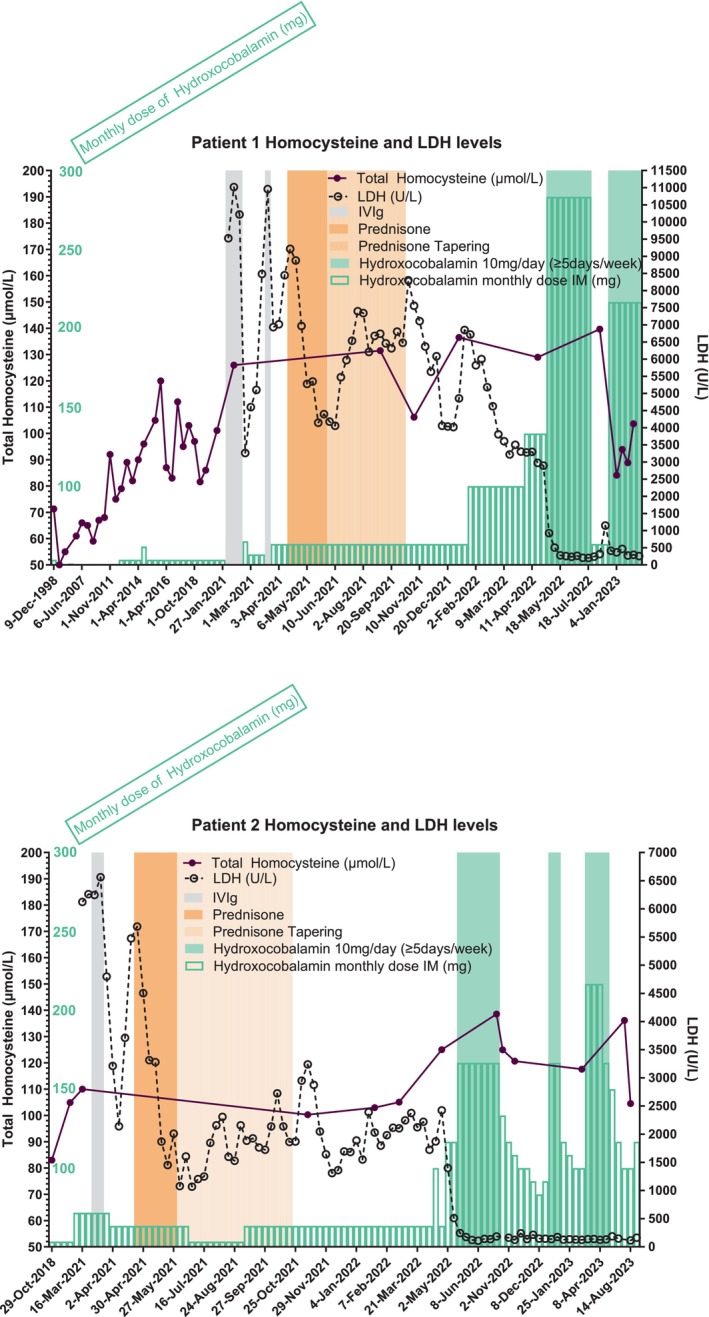
Plasma total homocysteine and LDH concentrations evolution over time and changes in hydroxocobalamin dosages. LDH, lactate dehydrogenase concentration in international units/L; IVIg, intravenous immunoglobulins 1 g/kg/day; Prednisone, 1 mg/kg/day; cumulative monthly dose of hydroxocobalamin is shown in green lined bars expressed in mg.

Biochemical monitoring was regularly scheduled every 3–6 months, which showed a stable but high plasma total homocysteine concentration even after they both were infected by SARS‐CoV2 in December 2021 with a pauci symptomatic infection (Figure 3). Non‐improvement of hematologic parameters along with imperfect control of metabolic parameters (high plasma total homocysteine and methionine plasma concentration remaining in the low‐normal range) were an incentive to propose major hydroxocobalamin dose escalations for the 2 brothers 12 months after the initial referral. Hydroxocobalamin was therefore administered intramuscularly daily at 10 mg (Figures 1 and 2). This regimen allowed for a drastic and complete normalization of LDH and hemoglobin level within a month, without any further transfusion (Figures 1 and 2). Furthermore, total homocysteine plasma concentration decreased belatedly 6 months after dosage modification of hydroxocobalamin (Figure 3).

## 2 DISCUSSION

This case series reports novel phenotypic features in cblE defect, that is, severe hemolysis that can be monitored and the possibility of parvovirus B19 infection as a trigger for acute hemolytic anemia in an immunocompetent adult with this inborn error of metabolism.

Anemia is the most frequent cytopenia in remethylation disorders and is thought to be related to the accumulation of intracellular 5‐methyltetrahydrofolate because of the decreased activity of MTR. This accumulation is regarded as a folate depletion effect leading to disturbance of rapidly proliferating tissues like bone marrow.^9^, ^10^ MTR activity can be lowered not only by methylcobalamin shortage but also with inhibiting agents, such as the anesthetic gas N_2_O through irreversible oxidation of cobalamin to Cbl‐III.^11^ Other molecules have been identified as inhibitors such as methylmercury, ethanol, or carbon tetrachloride (cleaning agent), which are important to look for in case of acute anemia as in our cases.^12^, ^13^

Regarding the hemolytic component of the anemia, it was demonstrated that oxidative stress can lead to deterioration of macromolecules of red blood cells (RBC) either on the membrane or in the cytoplasm, which ultimately leads to cell damage.^14^ In keeping with this, intracytoplasmic homocysteine in RBC could possibly form deleterious aggregates with cytoskeleton proteins between homocysteine's thiol group and sulfhydryl group RBC's proteins that could explain the hemolytic feature as well as abnormal ektacytometry in remethylation disorders during oxidative stress condition and glutathione deficiency.^15^, ^16^ Moreover, homocysteine association with membrane protein's sulfhydryl groups or their oxidation lead to accelerated RBC destruction in vivo, which would explain intramedullar hemolysis.^15^, ^17^ Homocysteine is recognized as a deleterious factor for RBC longevity due to its peroxidation capacity of membrane proteins but also for its role in lowering glutathione that acts as an oxidation defense system in RBC.^18^, ^19^, ^20^

Hallmarks of intramedullary hemolysis are usually represented by ineffective or dysplastic erythropoiesis, elevated unconjugated bilirubin, low haptoglobin, and increased LDH levels in plasma.^21^, ^22^ It is compatible with megaloblastic anemia due to impaired B12 metabolism. Interestingly, it has been reported in Biermer's anemia or other B12 deficiency conditions, the occurrence of hemolytic anemia similar to our cases.^23^, ^24^, ^25^

Intramedullary hemolysis can be enhanced by parvovirus B19 (PVB19) which directly targets erythroblasts in the bone marrow by interacting with the highly expressed globoside (P antigen) on erythroid precursors. Indeed, replication of this erythrovirus ultimately leads to giant pro‐erythroblasts that release infectious particles by bursting.^26^, ^27^ Diagnostic is easily evoked with rapidly progressive anemia and reticulocytopenia <20 G/L. Bone marrow aspiration confirms erythroblastopenia as well as giant proerythroblasts, and PCR for PVB19 confirms the diagnosis of infection with a higher sensitivity than blood PCR or serology.^28^, ^29^ Whereas PVB19 infection is a usual cause of aggravated hemolysis in patients with constitutional hemolysis, no case has been reported in remethylation disorders. Clinical signs present mostly 18–20 days after contamination. The prognosis is good in the general population without any intervention, whereas immunocompromised patients or those with RBC disease may require RBC transfusion or IVIg.^27^ Regarding our case reports, the causative role of PVB19 remains unclear as it was only found in one of the 2 brothers who both exhibited the same acute hemolysis scenario and the absence of improvement despite IVIg.

Another concern in this clinical picture is that anemia occurred despite a regular supplementation with intramuscular hydroxocobalamin and persisted after an increase in the dosing regimen with daily intramuscular injection. It required much higher doses of hydroxocobalamin to initiate a rapid response. The sudden loss of efficacy of low‐dose hydroxocobalamin could possibly advocate for an acquired additional abnormality. The manufacturer and pharmacy of hydroxocobalamin were contacted and ruled out any modification of production or incident on the chain of production or conservation. The hypothesis of anti‐transcobalamin II (TCBII) was raised since antibodies directed against Cbl‐II transporting protein have been identified in few cases after intramuscular cyanocobalamin supplementation.^30^, ^31^ However, data about their potential pathogenic effect are scarce and only reported in individuals without a remethylation disorder. It is supposed that they disrupt TCBII uptake by cells, thus disturbing Cbl metabolism and also TCBII's elimination through urine. As these antibodies are not routinely measured, we did not test them. The effect of steroids and high doses of cobalamin might have countered this blockade.

Regardless, it is plausible that both brothers required at the same age higher needs of hydroxocobalamin therapy to counteract bone marrow failure. Many centers use 1 mg of hydroxocobalamin in daily dose for neonates which is equivalent to 0.33 mg/kg assuming a body weight of 3 Kg before decreasing the frequency of administration.^1^, ^2^ However, 1 mg for adults would represent a tiny amount and might explain the lack of efficacy of low dose of hydroxocobalamin. A higher daily dose as in our case series might be necessary during growth or metabolic stress to overcome symptoms.

cblE patients are rarely reported in the literature and caution is needed regarding apparent novel clinical findings at adult age such as severe hemolysis. It would be worth looking at hemolytic markers in other types of remethylation disorders such as the more frequent cblC type, to confirm these findings. The use of LDH for monitoring might be another helpful laboratory parameter besides usual hematological and metabolic parameters to optimize hydroxocobalamin supplementation.

To conclude, the observation of novel hemolytic and infectious features in this rare disease should raise awareness about specific adulthood complications in remethylation disorders to optimize management, especially hydroxocobalamin dose escalation.

## CONFLICT OF INTEREST STATEMENT

The authors declare no conflicts of interest.

## ETHICS STATEMENT

This work was conducted in accordance with the principles of the Declaration of Helsinki. The participants gave informed consent for publication.

## References

[1] Huemer M , Baumgartner MR . The clinical presentation of cobalamin‐related disorders: from acquired deficiencies to inborn errors of absorption and intracellular pathways. J Inherit Metab Dis. 2019;42:686‐705. doi:10.1002/jimd.12012 30761552

[2] Huemer M , Diodato D , Schwahn B . Guidelines for diagnosis and management of the cobalamin‐related remethylation disorders cblC. J Inherit Metab Dis. 2017;40(1):21‐48. 27905001 10.1007/s10545-016-9991-4 PMC5203859

[3] Wolthers KR , Scrutton NS . Cobalamin uptake and reactivation occurs through specific protein interactions in the methionine synthase‐methionine synthase reductase complex. FEBS J. 2009;276:1942‐1951. doi:10.1111/j.1742-4658.2009.06919.x 19243433

[4] Zheng D , Yan L , Birke RL . Electrochemical and spectral studies of the reactions of aquocobalamin with nitric oxide and nitrite ion. Inorg Chem. 2002;41:2548‐2555. doi:10.1021/ic010802a 11978125

[5] Kandula T , Peters H , Fahey M . Cobalamin E defect, a rare inborn error of vitamin B12 metabolism: value of early diagnosis and treatment. J Clin Neurosci. 2014;21:1815‐1817. doi:10.1016/j.jocn.2013.12.030 24844621

[6] Zavadáková P , Fowler B , Zeman J , et al. CblE type of homocystinuria due to methionine synthase reductase deficiency: clinical and molecular studies and prenatal diagnosis in two families. J Inherit Metab Dis. 2002;25:461‐476. doi:10.1023/A:1021299117308 12555939

[7] Huemer M , Bürer C , Ješina P . Clinical onset and course, response to treatment and outcome in 24 patients with the cblE or cblG remethylation defect complemented by genetic and in vitro enzyme study data. J Inherit Metab Dis. 2015;38:957‐967. doi:10.1007/s10545-014-9803-7 25526710

[8] Schiff M , Benoist JF , Tilea B . Isolated remethylation disorders: do our treatments benefit patients? J Inherit Metab Dis. 2011;34:137‐145. 20490923 10.1007/s10545-010-9120-8

[9] Kutzbach C , Stokstad EL . Mammalian methylenetetrahydrofolate reductase. Partial purification, properties, and inhibition by S‐adenosylmethionine. Biochim Biophys Acta. 1971;250:459‐477. doi:10.1016/0005-2744(71)90247-6 4399897

[10] Scott JM , Weir DG . The methyl folate trap. A physiological response in man to prevent methyl group deficiency in kwashiorkor (methionine deficiency) and an explanation for folic‐acid induced exacerbation of subacute combined degeneration in pernicious anaemia. Lancet. 1981;2(8242):337‐340. 6115113 10.1016/s0140-6736(81)90650-4

[11] Nicolaou A , Kenyon SH , Gibbons JM . In vitro inactivation of mammalian methionine synthase by nitric oxide. Eur J Clin Invest. 1996;26:167‐170. doi:10.1046/j.1365-2362.1996.122254.x 8904527

[12] Alam RTM , Abu Zeid EH , Khalifa BA . Dietary exposure to methyl mercury chloride induces alterations in hematology, biochemical parameters, and mRNA expression of antioxidant enzymes and metallothionein in Nile tilapia. Env Sci Pollut Res Int. 2021;28:31391‐31402. doi:10.1007/s11356-021-13014-5 33606169

[13] Banks EC , Doughty SW , Toms SM . Inhibition of cobalamin‐dependent methionine synthase by substituted benzo‐fused heterocycles. FEBS J. 2007;274:287‐299. doi:10.1111/j.1742-4658.2006.05583.x 17222188

[14] Caprari P , Bozzi A , Malorni W . Junctional sites of erythrocyte skeletal proteins are specific targets of tert‐butylhydroperoxide oxidative damage. Chem Biol Interact. 1995;94:243‐258. doi:10.1016/0009-2797(94)03339-a 7820887

[15] Ohmiya Y , Nakai K . Relationship between inhibition of membrane SH groups and hemolysis induced by SH inhibitors. Jpn J Pharmacol. 1977;27:596‐599. doi:10.1254/jjp.27.596 926465

[16] Vayá A , Bonet E , Romagnoli M . Erythrocyte deformability in macrocytosis determined by means of ektacytometry techniques. Clin Hemorheol Microcirc. 2010;45:27‐33. doi:10.3233/CH-2010-1284 20571227

[17] Ventura P , Panini R , Tremosini S , Salvioli G . A role for homocysteine increase in haemolysis of megaloblastic anaemias due to vitamin B12 and folate deficiency: results from an in vitro experience. Biochim Biophys Acta. 2004;1739:33‐42. doi:10.1016/j.bbadis.2004.08.005 15607115

[18] Dumaswala UJ , Zhuo L , Mahajan S . Glutathione protects chemokine‐scavenging and antioxidative defense functions in human RBCs. Am J Physiol Cell Physiol. 2001;280:C867‐C873. doi:10.1152/ajpcell.2001.280.4.C867 11245604

[19] Góth L , Vitai M . The effects of hydrogen peroxide promoted by homocysteine and inherited catalase deficiency on human hypocatalasemic patients. Free Radic Biol Med. 2003;35:882‐888. doi:10.1016/s0891-5849(03)00435-0 14556852

[20] van Zwieten R , Verhoeven AJ , Roos D . Inborn defects in the antioxidant systems of human red blood cells. Free Radic Biol Med. 2014;67:377‐386. doi:10.1016/j.freeradbiomed.2013.11.022 24316370

[21] Aslinia F , Mazza JJ , Yale SH . Megaloblastic anemia and other causes of macrocytosis. Clin Med Res. 2006;4:236‐241. doi:10.3121/cmr.4.3.236 16988104 PMC1570488

[22] McCarthy CF , Fraser ID , Read AE . Plasma lactate dehydrogenase in megaloblastic anaemia. J Clin Pathol. 1966;19:51‐54. doi:10.1136/jcp.19.1.51 5904983 PMC473158

[23] De La Puerta R , Carpio N , Sanz G , Solves P . A case of megaloblastic anemia simulating a cold autoimmune hemolytic anemia. Immunohematology. 2020;36:89‐92. 33112632

[24] Federici L , Henoun Loukili N , Zimmer J . Update of clinical findings in cobalamin deficiency: personal data and review of the literature. Rev Med Interne. 2007;28:225‐231. doi:10.1016/j.revmed.2006.10.319 17141377

[25] Woodford AM , Chaudhry R , Conte GA . Chronic atrophic gastritis presenting as hemolytic anemia due to severe vitamin B12 deficiency. Case Rep Hematol. 2021;2021:1‐5. doi:10.1155/2021/9571072 PMC8349249 34373795

[26] Heegaard ED , Brown KE . Human parvovirus B19. Clin Microbiol Rev. 2002;15:485‐505. doi:10.1128/CMR.15.3.485-505.2002 12097253 PMC118081

[27] Sim JY , Chang L‐Y , Chen J‐M . Human parvovirus B19 infection in patients with or without underlying diseases. J Microbiol Immunol Infect. 2019;52:534‐541. doi:10.1016/j.jmii.2019.05.009 31257106

[28] Iwantschenko A‐K , Roegener F , Garrels W . Why serology just is not enough: strategic parvovirus risk assessment using a novel qPCR assay. Lab Anim. 2022;56:380‐395. doi:10.1177/00236772211062861 35102773

[29] Jordan JA . Diagnosing human parvovirus B19 infection: guidelines for test selection. Mol Diagn J Devoted Underst Hum Dis Clin Appl Mol Biol. 2001;6:307‐312. 10.1054/modi.2001.28632 11774195

[30] Carmel R , Tatsis B , Baril L . Circulating antibody to transcobalamin II causing retention of vitamin B12 in the blood. Blood. 1977;49(6):987‐1000. 861380

[31] de Haro L , Marquet J , Tonetti C , Zittoun J . Hypervitaminémie B12 sérique due à un anticorps antitranscobalamine II: à propos d'un cas. Rev Médecine Interne. 2001;22:1132‐1133. doi:10.1016/S0248-8663(01)00481-7 11817129

